# Sex differences in the modulation of anxiety- and depression-like behaviors by matrix metalloproteinase-9 expression levels in mice

**DOI:** 10.1186/s13293-025-00716-5

**Published:** 2025-05-22

**Authors:** Júlia Senserrich, Elena Castro, Eva Florensa-Zanuy, Álvaro Díaz, Ángel Pazos, Albert Adell, Athina Tzinia, Fuencisla Pilar-Cuéllar

**Affiliations:** 1 Departamento de Señalización Molecular y Celular, Instituto de Biomedicina y Biotecnología de Cantabria (IBBTEC), IBBTEC (Universidad de Cantabria, CSIC, SODERCAN), Avda. Albert Einstein, 22, Santander, 39011 Spain; 2 Centro de Investigación Biomédica en Red de Salud Mental (CIBERSAM), Instituto de Salud Carlos III, Santander, Spain; 3 Departamento de Fisiología y Farmacología, Facultad de Medicina, Universidad de Cantabria, Santander, Spain; 4 Institute of Biosciences and Applications, National Center for Scientific Research “Demokritos”, Agia Paraskevi, Athens, Greece

**Keywords:** Depression, Anxiety, Matrix metalloproteinase-9Transgenic mice, Sex, Neuroplasticity

## Abstract

**Background:**

Major depressive disorder is one of the main causes of disability worldwide, but its etiopathology remains largely unknown, although several hypotheses have been proposed. Recent studies suggest a potential role for matrix metalloproteinase 9 (MMP-9) in depression, as it is overexpressed in the plasma of depressed patients and normalizes following chronic antidepressant treatment. This study aimed to characterize anxiety and depression-like behaviors in transgenic MMP-9 mice, as well as the expression of different neuroplasticity markers associated with depression, in both sexes.

**Methods:**

In this study, we characterized the behavioral phenotypes of both MMP-9 knockout and MMP-9-overexpressing male and female mice. Here, we used a battery of tests to assess anxiety (open field, light‒dark box, elevated plus maze, and novelty‒suppressed feeding tests), depressive-like (tail suspension and social interaction tests), and cognitive (T-maze) behaviors.

**Results:**

MMP-9 knockout female mice displayed increased innate anxiety (open field test), decreased behavioral despair (tail suspension test). Compared with control mice, female MMP-9 knockout mice presented increased levels of different neuroplasticity markers in the hippocampus. With respect to MMP-9-overexpressing mice, females presented decreased innate anxiety (elevated plus maze). Male MMP-9-overexpressing mice presented greater conflict-based anxiety (novelty-suppressed feeding test) than control mice did.

**Conclusions:**

MMP-9 activity modifies anxiety- and depression-like behaviors, as well as neuroplasticity markers, in female but not in male mice. These findings reinforce the sex differences in the etiopathology of depression.

**Supplementary Information:**

The online version contains supplementary material available at 10.1186/s13293-025-00716-5.

## Highlights

MMP-9 overexpressing female mice display low anxiety.

MMP-9 knockout female mice display low depressive-like behavior.

MMP-9 knockout female mice show higher hippocampal neuroplasticity markers expression.

MMP-9 expression levels do not have an impact on mood behavior and neural plasticity in male mice.

**Supplementary Information:**

The online version contains supplementary material available at 10.1186/s13293-025-00716-5.

## Abstract (significance)

Major depressive disorder is one of the most common neuropsychiatric diseases, which affects a two-fold number of women than men. The cause of this disorder is not known, however, impairments in neurotransmitters and neuroplasticity are some of the suggested causes. Specifically, some studies indicate that the expression of the matrix metalloproteinase 9 (MMP-9), one protein implicated in neuroplasticity, is increased in patients with depression, and these levels are reduced after antidepressant treatment. However, to date it has not been studied the differential implication of MMP-9 in the sex-differences observed in this pathology. In our study, we evaluated the impact of the elimination or the overexpression of MMP-9 in anxious and depressive-like behavior in male and female mice. We also evaluated how neuroplasticity was affected in these animals. We observed in female mice an opposite effect to the expected phenotype regarding anxiety, with higher anxiety in mice lacking MMP-9 and lower anxiety in mice that overexpress this protein. Moreover, the lower behavioral despair expected in mice that lack MMP-9 was only observed in females, which was parallel to the higher expression of some neuroplasticity markers. In conclusion, we demonstrate that MMP-9 activity affects mood behavior, as well as neuroplasticity, in female but not in male mice.

**Supplementary Information:**

The online version contains supplementary material available at 10.1186/s13293-025-00716-5.

## Background

Major depressive disorder (MDD) is a chronic psychiatric condition characterized by low mood, anhedonia, psychomotor alterations, feelings of guilt, and suicidal ideation. Patients can also present cognitive impairment and reduced libido [1]. According to the World Health Organization (WHO), MDD affects 3.8% of the population, which includes 5% adults, and has a higher prevalence in women than in men [2]. Moreover, the WHO reported that the COVID-19 pandemic induced a 25% increase in depression and anxiety cases [3].

The etiopathogenesis of MDD is not fully known, although multiple risk factors are involved, including biological, genetic, and psychosocial factors. Since the formulation of the classical “monoaminergic” hypothesis [4], which proposes the existence of a dysfunctional brain monoaminergic system associated with depression, different hypotheses, including the *neuroplastic*,* glutamatergic*, and *neuroinflammatory* hypotheses, have been proposed to explain the neurobiology of this disease. Understanding the mechanisms underlying the pathophysiology of MDD is highly important for the development of novel therapeutic approaches.

Matrix metalloproteinases (MMPs) are endopeptidases responsible for the cleavage of the extracellular matrix and basement membrane components, cell surface receptors, cell adhesion molecules, growth factors, cytokines, and other proteases. Interestingly, they act as regulators of not only extracellular but also intracellular signaling networks [5, 6]. One of the most studied MMPs is MMP-9, a gelatinase expressed in both the peripheral and central nervous systems. It is released from glutamatergic excitatory synapses upon the activation of NMDA receptors [7]. MMP-9 plays a pivotal role in the regulation of synaptic plasticity, learning and memory, allowing the growth and maturation of dendritic spines and the accumulation and immobilization of AMPA receptors, which makes excitatory synapses more efficient in modulating the AMPA/NMDAR ratio [8, 9]. Moreover, MMP-9 can catalyze the activation of brain-derived neurotrophic factor (BDNF) and proinflammatory cytokines, which participate in the processes of neurogenesis and inflammation, respectively [7]. All these biological functions explain the involvement of MMP-9 in the etiopathogenesis of certain neurodegenerative disorders, such as epilepsy [10] and multiple sclerosis [11].

MDD patients present increased blood levels of the matrix metalloproteinases MMP-2, MMP-7, and MMP-9 [12–14]. Serum MMP-9 levels are positively correlated with the severity of symptoms [15–18]. In addition, electroconvulsive therapy reduces the serum levels of MMP-9 in depressed patients, resulting in a therapeutic response [19]. Increased MMP-9 activity has also been shown in *postmortem* hippocampal samples from MDD patients [20], whereas other authors reported no differences in MMP-9 protein levels in the prefrontal cortex of untreated MDD patients [21]. Moreover, different MMP-9 gene polymorphisms, which are associated with high MMP-9 levels, have been associated with depressive symptoms in bipolar disorder patients (rs17576) [22] and anxiety disorders (rs3918242) [23]. These findings highlight the importance of MMP-9 as a potential diagnostic marker of depression [24], which is correlated with treatment response and disease progression.

Regarding the role of brain MMP-9 in animal models of depression, we recently reported increased expression and activity of MMP-9 in the hippocampus and cortex of a chronic corticosterone mouse model of depression [25]. Similar results have been shown in other models of depression, such as the chronic stress model [26], the chronic unpredictable stress model (CUS) in mice [20], and a neuroinflammation model [27].

In this study we evaluate different molecular markers in the hippocampus. The hippocampus is one of the few brain regions extensively involved in neurogenesis, and the dysregulation of adult hippocampal neurogenesis has been reported in major depressive disorder [28]. Additionally, the hippocampus plays a crucial role in anxiety regulation due to its connections with regions such as the amygdala and the cingulate cortex [29, 30]. Several proteins related to neuroplasticity processes have been found to be altered in major depression [31] and animal models of depression [32], and restored by antidepressant drugs, including mTOR [32], PSD95 [33], BDNF [34, 35] and synapsin I [33, 36].

In the last few years, the use of female animal models has increased, supporting the urge to address preclinical research to define optimal female models of neuropsychiatric diseases and their use for a deeper knowledge of sex bias. Moreover, a better understanding of the role of MMP-9 in the development of a depressive/anxious-like phenotype would help to elucidate the role of this protein in the neurobiology of depression. Therefore, in this study, we characterized the anxious and depressive-like behavioral and molecular phenotypes of male and female mice of two transgenic models: MMP-9 knockout (MMP-9 KO) mice, which have a nonfunctional MMP-9 protein, and mice that overexpress the human MMP-9 (MMP-9 OE) in the central nervous system.

## Methods

### Animals

The MMP-9 knockout (Jackson Laboratory, Maine, USA) and MMP-9 overexpression (kindly donated by Dr. A.K. Tzinia) male and female mice, 2–3 months old, were group-housed (4–5 mice per cage) with a 12 h light‒dark cycle and food and water *ad libitum*. All procedures were carried out with the previous approval of the Animal Care Committee of the University of Cantabria and according to Spanish legislation (RD 53/2013) and the European Communities Council Directive on “Protection of Animals Used in Experimental and Other Scientific Purposes” (2010/63/UE).

#### MMP-9 knockout mice

B6.FVB(Cg)-Mmp9tm1Tvu/J (MMP-9 KO) mice (#007084, The Jackson Laboratory, Maine, USA) were generated by inserting a neomycin resistance gene driven by the mouse phosphoglycerate kinase promoter. This cassette replaces most of exon 2 and all of intron 2, disrupting MMP-9 functionality. This transgenic mouse line was originated on a B6;129 background, they were crossed to Black Swiss mice and then to FVB/N mice. Finally, it was crossed to C57BL/6J mice for more than 10 generations.

#### MMP-9-overexpressing mice

TgMMP9 (MMP-9 OE) mice were generated with the PDGF-B/MMP9 transgene of human origin, a gene encoding human pro-MMP-9 under the control of the human neuron-specific PDGF-B promoter. The founders were of a B6;DBA background, and then crossed with C57BL/6 for more than 10 generations [37].

### Behavioral tests

**Fig. 1:**
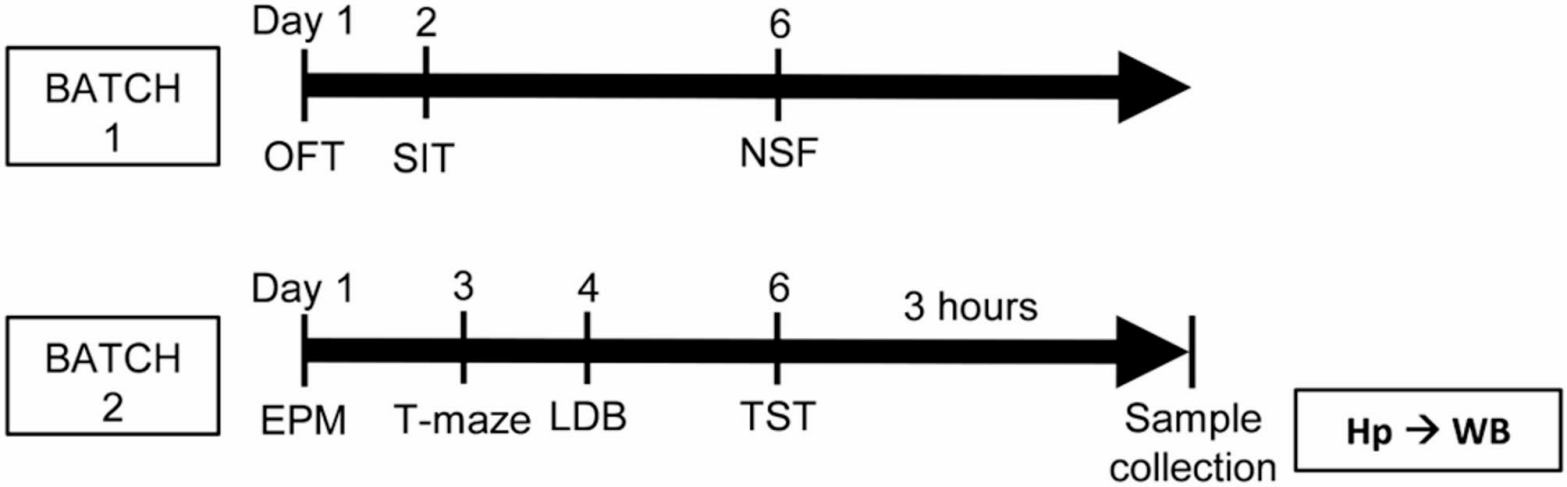
Experimental timeline for behavioral tests. EPM: elevated plus maze; LDB: light-dark box; NSF: novelty-suppressed feeding; OFT: open field test; SIT: social interaction test; TST: tail suspension test; Hp: hippocampus; WB: Western blot

All the behavioral tests were performed during the light phase. Female mice were not monitored for estrous cycle stage to avoid an additional source of stress taking vaginal smears. Mice were placed in the experimental room one hour before the evaluation to allow them to acclimatize. Two different batches of animals were used to avoid an overload of stress due to the battery of behavioral tests assessed (Fig. 1). Three hours after the last experiment, the mice were sacrificed via cervical dislocation. The brains were rapidly removed, and the hippocampus was dissected on an ice-cold platform and kept at -80 °C until use for western blot experiments.

#### Open field test (OFT)

The OFT was performed as previously described by Breviario et al. [25]. Mice were placed in a corner of the arena (40 × 40 × 30 cm) with an illuminated (50 lx) center (20 × 20 cm). The central time and total distance traveled were video tracked (Any-Maze™ tracking software version 4.99; Stoelting Co., Wood Dale, USA) for 5 min.

#### Elevated plus maze (EPM)

Mice were placed in the center (5 × 5 cm) of the apparatus (50 cm from the floor), which consists of two open arms (25 × 5 × 0.5 cm) and two perpendicular closed arms (25 × 5 × 16 cm). Mice were allowed to explore freely for 5 min. The time spent in each arm was video tracked (Any-Maze™).

#### Light-Dark box (LDB)

The apparatus consists of a square box (40 × 40 × 20 cm) divided into two halves, one illuminated (50 lx) area and one dark area, connected by an opening. Mice were placed in a corner of the illuminated compartment, and the animal was allowed to freely explore the apparatus [38]. The time spent in each area was video tracked (Any-Maze™) for 5 min.

#### Novelty-suppressed feeding (NSF) test

NSF was performed as previously described by Vidal et al. [38]. Mice were food deprived for 24 h. A food pellet was placed in the illuminated center (50 lx) of an arena (40 × 40 × 30 cm) covered with a woodchip. The mouse was placed in a corner, and the latency to eat the pellet was recorded via video-tracking software (Any-Maze™) for a maximum duration of 10 min. After the test, the mice were placed back in their home cages, and the amount of food consumed for 5 min was measured. The animals that showed no food intake in their home cages were excluded from the data analysis.

#### Tail suspension test (TST)

Mice were suspended by the tail for 6 min. Video-tracking software (Any-Maze™) was used to record the test results, and the time spent immobile was determined manually for the last 4 min by an observer blinded to the experimental groups.

#### Social interaction test (SIT)

Mice were placed in an open field arena (40 × 40 × 30 cm) with an empty wire cylinder (7 cm diameter) placed in a corner. The experimental mice were allowed to explore the arena freely for 2.5 min. Immediately after, an unfamiliar mouse of a different strain (CD-1) was placed inside the wire cylinder, and the experimental mice were allowed to explore for 2.5 min. The time in the interaction zone (20 cm diameter) was assessed via video-tracking software (Any-Maze™).

#### T-maze

The T-maze was adapted from a previously described protocol [39]. The apparatus consists of three arms, two opposite (30 × 5 × 15 cm) and one perpendicular (35 × 5 × 15 cm), creating a “T” shape. First, a training trial was assessed in which the left arm of the T-maze was blocked. The mice were placed at the end of the perpendicular arm and allowed to explore for 8 min. After the training trial, mice were removed from the apparatus and left to rest for an intertrial interval of 1 h. Then, for the experimental trial, the left arm was unlocked, recovering the “T” shape. A 3 min test was performed by placing the mice at the end of the perpendicular arm and allowing them to freely explore the apparatus. Mice were video tracked (Any-Maze™), and the time spent in the novel arm during the first minute of the test was measured.

### Z-Score and emotionality

The individual z-scores of the time spent in the center of the OFT, time spent in the open arms of the EPM, time spent in the light zone of the LDB, and the latency to feeding of the NSF (anxiety parameters), and immobility time in the TST and the time in the interaction zone in the SIT (depression parameters), were calculated using the following formula [40]: z=(X-µ)/σ, where X is the individual data, µ is the mean of the male and female WT mice, and σ is the standard deviation of µ. The symbol of the z-scores was inverted in the OFT, EPM, LDB, and SIT, so that a positive score represents an increase in the anxiety- or depressive-like phenotype, and vice versa. Then, individual emotionality scores were calculated as the mean of the z-scores in the anxious-related tests of the corresponding batch. Finally, the mean z-emotionality for anxiety and depression were obtained integrating the individual emotionality of the anxiety-like behavior and the z-scores of the depressive-like behavior.

### Western blot

#### Synaptoneurosomal protein extraction

Protein extraction was performed according to the protocol previously described by Li et al. [41]. Briefly, the hippocampi were homogenized (1:15 w/v) in homogenization buffer (0.32 M sucrose, 20 mM HEPES pH 7.4, 1 mM EDTA, 1:100 protease inhibitor cocktail, 5 mM NaF and 1 mM Na_3_VO_4_). The samples were subsequently centrifuged at 800xg for 10 min at 4 °C. The pellet was removed, and the supernatant was centrifuged at 15 300xg for 10 min at 4 °C. The pellet was resuspended in 150 µl of protein lysis buffer (50 mM Tris-HCl pH 7.5, 150 mM NaCl, 1% Triton X-100, 0.1% SDS, 2 mM EDTA, 1 mM Na_3_VO_4_, 5 mM NaF and 1:100 protease inhibitor cocktail) and sonicated for 1 min. The protein concentration was quantified via the Lowry method. The samples were prepared with loading buffer containing β-mercaptoethanol, boiled at 100 °C for 5 min, cooled on ice for 3 min, and centrifuged at 1000xg for 5 min at 4 °C. The supernatant was stored at -20 °C until use.

#### Western blot

Male MMP-9 KO and their respective wildtypes, as well as female MMP-9 KO and their respective wildtypes, were loaded onto separate membranes simultaneously in the same experimental day. For MMP-9 OE, male and female samples, and their respective wildtype counterparts, were set following the same procedure as for MMP-9 KO mice. Fifty µg of protein per sample was loaded in duplicate on 8.5% or 15% SDS‒PAGE gels. The electrophoresis mixture was run at 100 V for 15 min and then at 160 V for 50 min and then transferred to nitrocellulose membranes (GE Healthcare Europe GmbH, Munich, Germany). The membranes were blocked with 5% (w/v) powder skim milk in TBS-T (50 mM Tris‐HCl pH 7.6, 150 mM NaCl, 0.05% Tween‐20) for 1 h. For phosphorylated proteins, the blocking solution used was 3% powder skim milk in TBS‐T supplemented with phosphatase inhibitors (1 mM Na_3_VO_4_ and 1 mM NaF). The following primary antibodies were incubated with the corresponding blocking solution at 4 °C overnight: BDNF (1:500, ab108319, Abcam, Cambridge, MA, USA), mTOR (1:1000, #4517, Cell Signaling, Massachusetts, USA), phospho-Ser2448-mTOR (1:1000, #2971, Cell Signaling, Massachusetts, USA), PSD95 (1:200, sc-8575, Santa Cruz Biotechnology, Texas, USA), synapsin I (1:200, sc-390867, Santa Cruz Biotechnology, Texas, USA), and β-tubulin III (1:20000, T2200/T8660, Sigma‒Aldrich, Missouri, USA). The specificity of the antibodies used in this study is shown in Table S1. The membranes were washed with TBS-T and then incubated with the corresponding secondary antibody conjugated to an NIR fluorophore (1:15 000, LI-COR Biosciences, Lincoln, NE, USA) for 1 h at room temperature. After washing, the specific signal was visualized via an Odyssey^®^ CLx Imaging System (LI-COR Bioscience, Lincoln, USA) and quantified via Image Studio™ Software (LI‐COR Biosciences, Lincoln, USA). The densitometric values were normalized to those of the housekeeping protein β‐tubulin III. The analysis of the β-tubulin expression levels did not present any differences between the experimental groups used in this study.

### Statistical analysis

The values are expressed as the means ± standard errors of the means (S.E.M.). For the molecular studies, the data were normalized to the housekeeping gene for each well, the mean of each duplicate was calculated, and the results were expressed as a percentage, with wildtype animals set at 100%. The data were analyzed via two-way ANOVA followed by Tukey’s *post hoc* test. The statistical analyses and identification of outliers were performed via GraphPad Prism 10 (GraphPad Software, Inc., California, USA). Statistical significance was set at *p* < 0.05.

## Results

### Innate anxiety in MMP-9 KO and OE mice

**Fig. 2:**
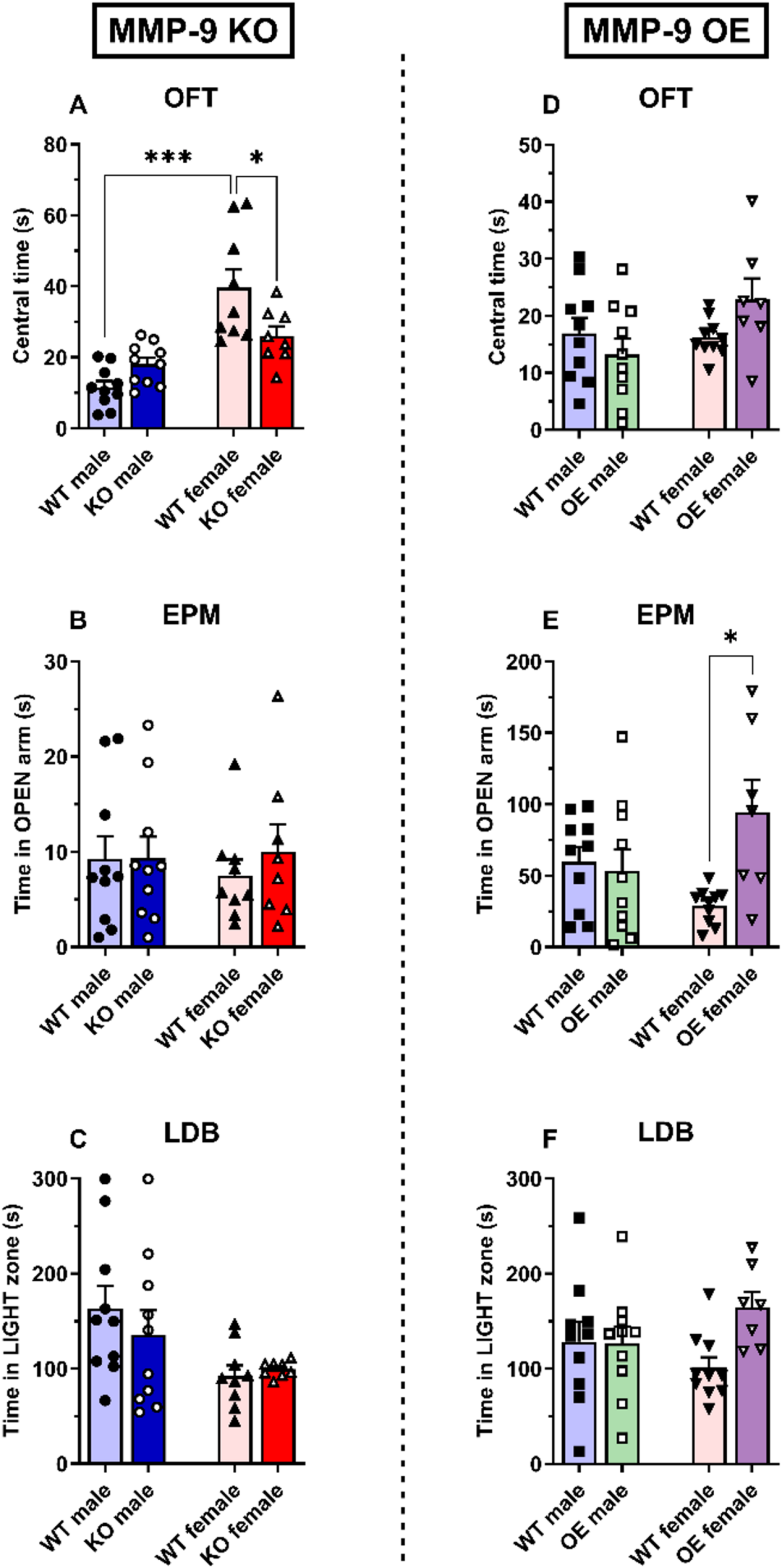
Innate anxiety in MMP-9 KO (**A**–**C**) and MMP-9 OE (**D**–**F**) male and female mice. Time spent in the center (**A** and **D**) in the open field test, time spent in the open arms in the elevated plus maze (**B** and **E**), and time spent in the lit compartment in the light-dark box test (**C** and **F**). The data are expressed as the means ± SEMs. Two-way ANOVA followed by a Tukey’s *post hoc* test. *p < 0.05 and ***p < 0.001. *n* = 7–10 animals per group. OFT: open field test; EPM: elevated plus maze; LDB: light‒dark box test. WT: wild-type mice; KO: MMP-9 knockout mice; OE: MMP-9-overexpressing mice

In the open field test (OFT), MMP-9 KO female mice spent less time in the center than WT female mice (*p* < 0.05), with no differences in MMP-9 KO male mice (Fig. 2A). Compared with male WT mice, female WT mice spent more time in the center (*p* < 0.001). Two-way ANOVA revealed a significant effect of sex [F(1,33) = 33.4, *p* < 0.001] and the interaction sex × genotype [F(1,33) = 10.4, *p* < 0.01].

In the OFT, MMP-9 OE male and female mice did not significantly differ in the time spent in the center compared with their respective WT mice (Fig. 2D). Two-way ANOVA revealed a trend toward the interaction sex × genotype [F(1,33) = 3.95, *p* = 0.06].

In the elevated plus maze (EPM) and light‒dark box (LDB) tests, no significant differences were found in the time spent in the open arms in the EPM (Fig. 2B) or the time spent in the light zone of the LDB (Fig. 2C) in male and female MMP-9 KO mice, compared to their WT counterparts. In the LDB test, two-way ANOVA revealed a significant effect of sex [F(1,33) = 7.2, *p* < 0.05].

In the EPM test, MMP-9 OE female mice spent more time in the open arms (*p* < 0.05, Fig. 2E), while no significant differences were found in the time spent in the light zone of the LDB test (Fig. 2F). There were no differences in MMP-9 OE male mice in either test. In the EPM test, a two-way ANOVA revealed a significant effect of the genotype [F(1,33) = 4.9, *p* < 0.05] and the interaction sex × genotype [F(1,33) = 7.2, *p* < 0.05].

### Conflict-based anxiety in MMP-9 KO and OE mice

**Fig. 3:**
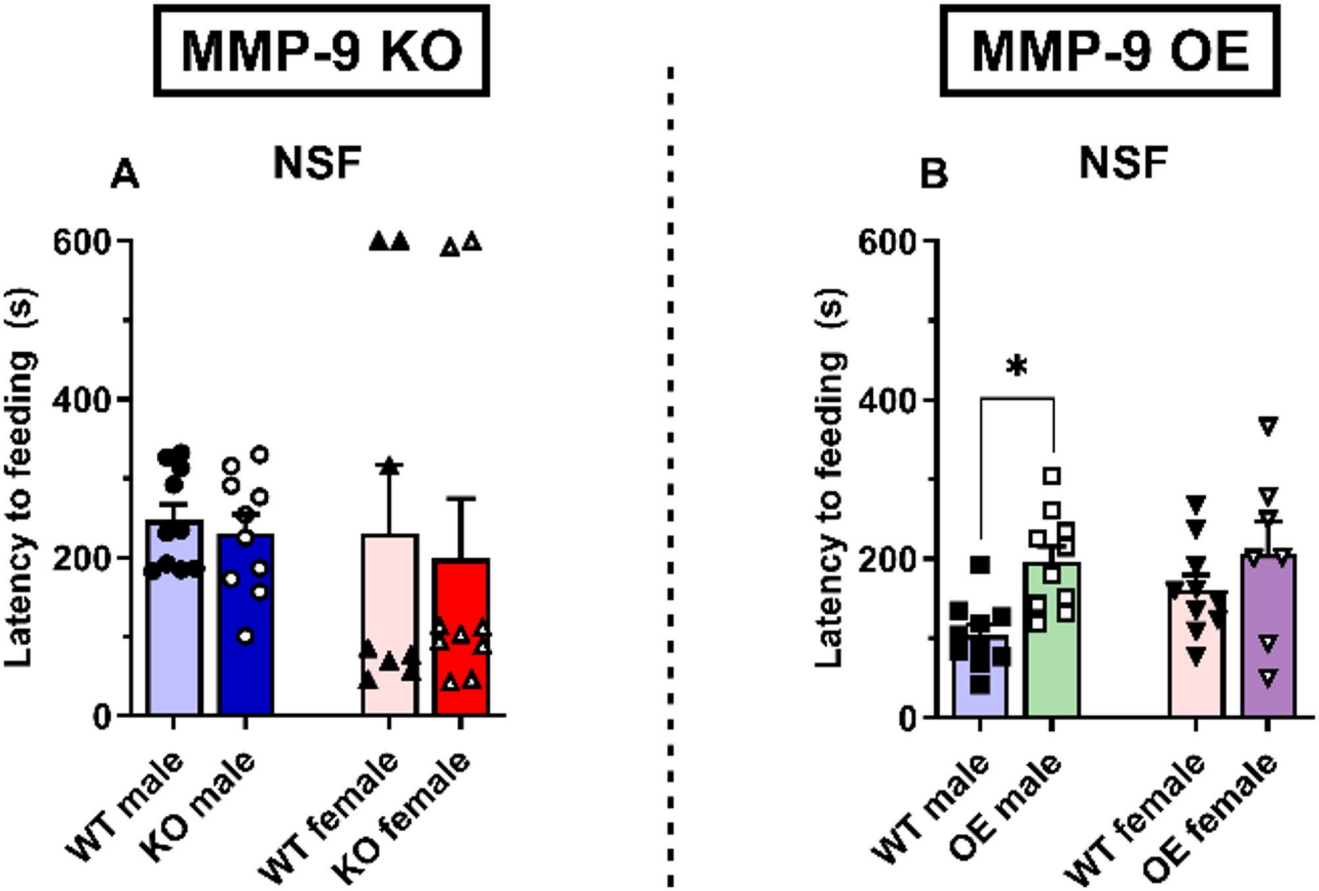
Conflict-based anxiety in MMP-9 KO (**A**) and MMP-9 OE (**B**) male and female mice assessed by the latency to feeding in the novelty-suppressed feeding test. The data are expressed as the means ± SEMs. Two-way ANOVA followed by a Tukey’s *post hoc* test. *p < 0.05. *n* = 7–10 animals per group. WT: wild-type mice; KO: MMP-9 knockout mice; OE: MMP-9-overexpressing mice

In the novelty-suppressed feeding (NSF) test, no significant differences were observed in the latency to feed in the male and female MMP-9 KO mice (Fig. 3A) and OE female mice (Fig. 3B). However, male MMP-9 OE mice presented a greater latency to feed (*p* < 0.01, Fig. 3B). Two-way ANOVA revealed a significant effect of genotype [F(1,33) = 9.4, *p* < 0.01]. Additionally, the amount of food consumed after the test did not significantly differ (see Figure S1).

### Depressive-like behavior in MMP-9 KO and OE mice

**Fig. 4:**
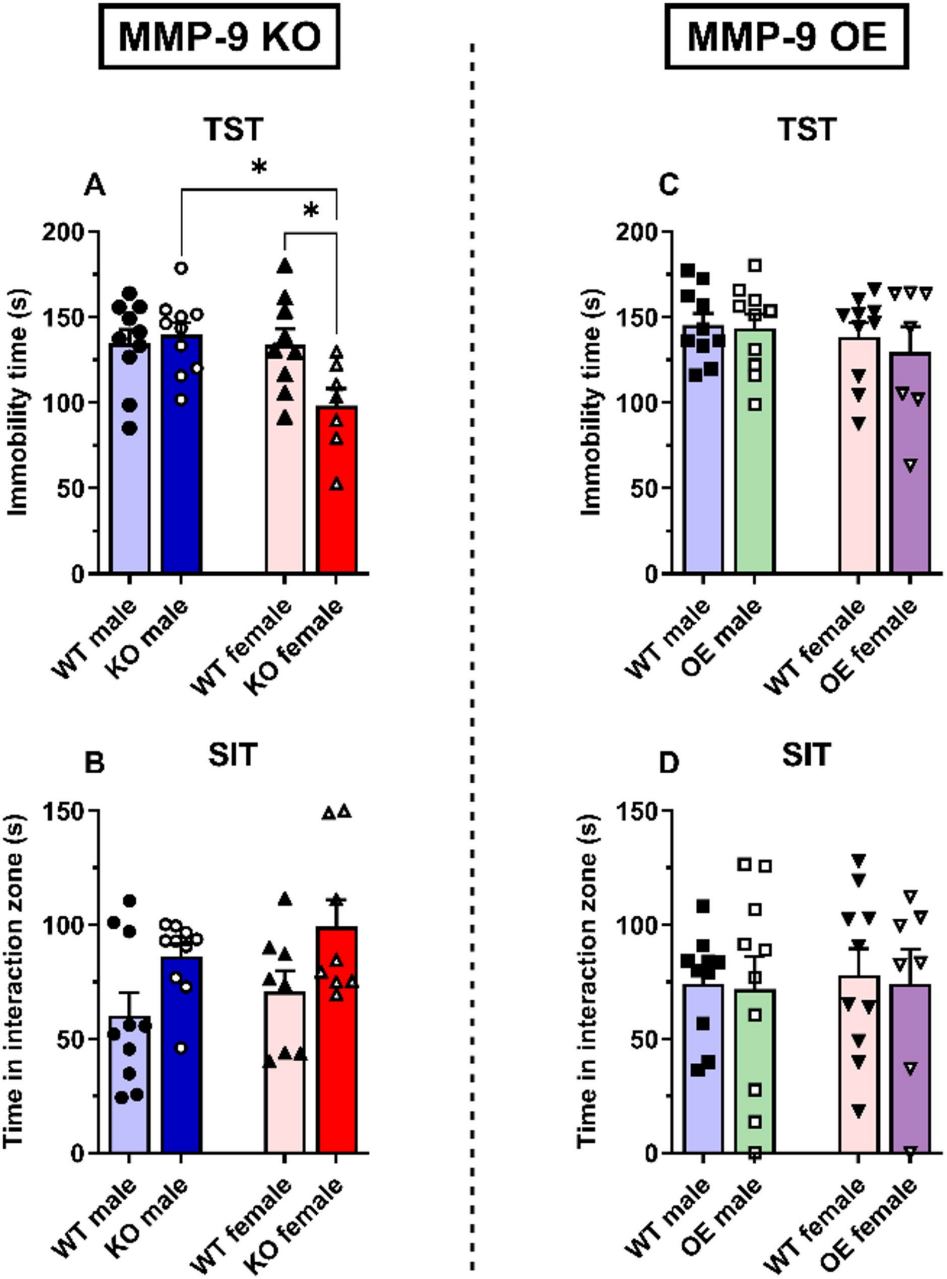
Depressive-like behavior in MMP-9 KO (**A** and **B**) and MMP-9 OE (**C** and **D**) male and female mice. Immobility time in the tail suspension test (**A** and **C**) and social interaction time (**B** and **D**) in the social interaction test. The data are expressed as the means ± SEMs. Two-way ANOVA followed by a Tukey’s *post hoc* test. *p < 0.05. *n* = 7–10 animals per group. TST: tail suspension test; SIT: social interaction test; WT: wild-type; KO: MMP-9 knockout; OE: MMP-9-overexpressing

In the tail suspension test (TST), MMP-9 KO female mice displayed a lower immobility time than WT female and MMP-9 KO male mice (*p* < 0.05, Fig. 4A). No differences were observed in the MMP-9 KO males (Fig. 4A). Two-way ANOVA revealed a significant effect of sex [F(1,32) = 6.0, *p* < 0.05] and the interaction sex × genotype [F(1,32) = 5.6, *p* < 0.05]. Both male and female MMP-9 OE mice did not significantly differ (Fig. 4D).

In the social interaction test (SIT), male and female MMP-9 KO mice did not display significant differences in sociability (Fig. 4B). Two-way ANOVA revealed a significant effect of genotype on MMP-9 KO mice [F(1,32) = 8.7, *p* < 0.01]. Compared with their WT counterparts, male and female MMP-9 OE mice did not significantly differ (Fig. 4D).

### Z-emotionality in MMP-9 KO and OE mice

**Fig. 5:**
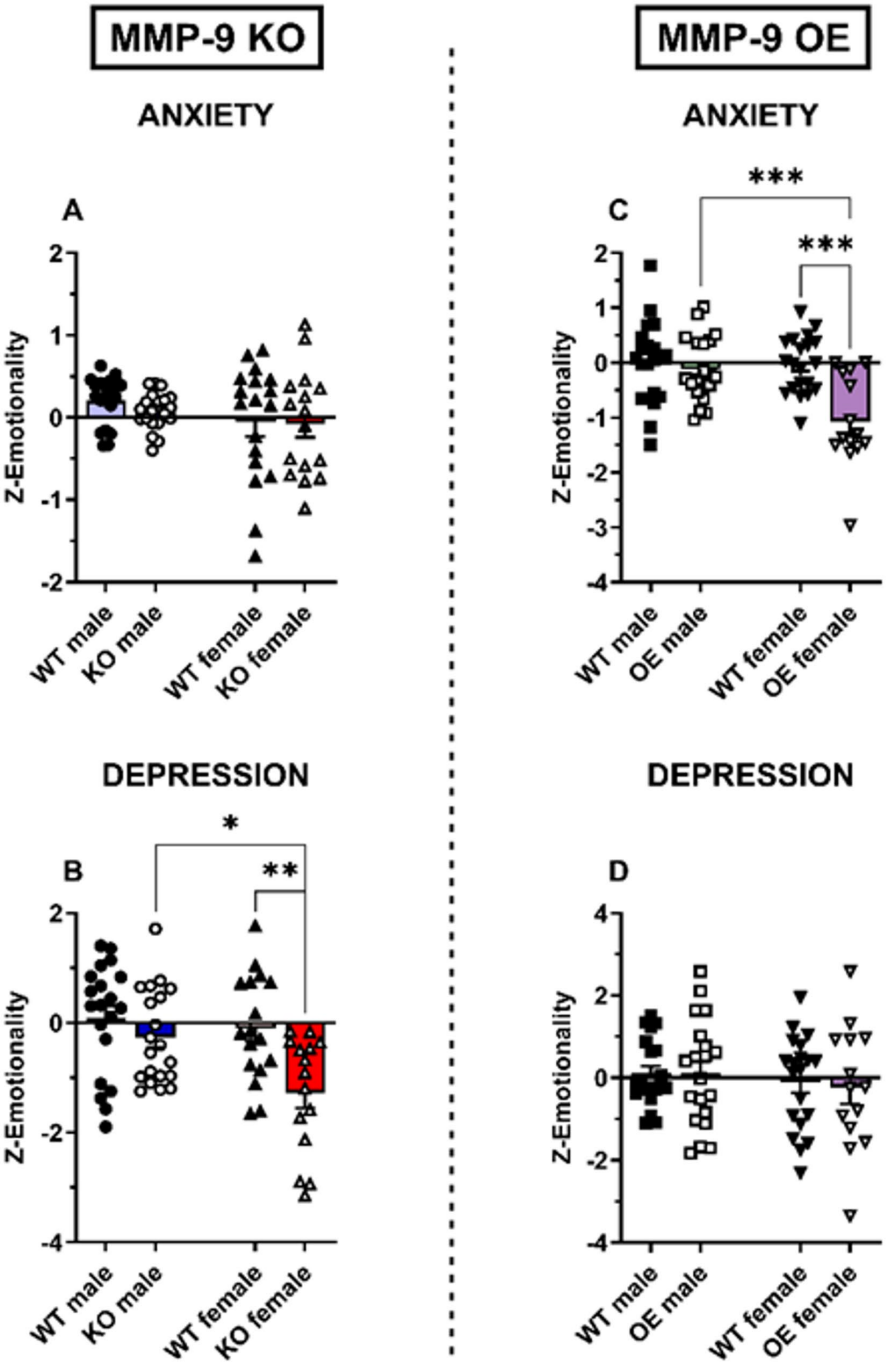
Z-emotionality scores in MMP-9 KO (**A** and **B**) and MMP-9 OE (**C** and **D**) male and female mice. Z-emotionality of anxiety (**A** and **C**) and of depression (**B** and **D**). The results are expressed as the means ± S.E.M. Two-way ANOVA followed by a Tukey’s *post hoc* test. *p < 0.05, **p < 0.01 and ***p < 0.001. *n* = 14-20 animals per group. WT: wild-type mice; KO: MMP-9 knockout mice; OE: MMP-9-overexpressing mice

The z-emotionality score in anxious-like behavior did not show significant differences between male and female MMP-9 KO mice and their WT counterparts (Fig. 5A). MMP-9 OE female group presented a lower score in anxiety-like behavior than WT female (*p* < 0.001) and MMP-9 OE male mice (*p* < 0.001, Fig. 5C). Two-way ANOVA revealed a significant effect of sex [F(1,70) = 10.0, *p* < 0.01], genotype [F(1,70) = 13.8, *p* < 0.001] and the interaction sex × genotype [F(1,70) = 8.0, *p* < 0.01].

The z-emotionality score in depressive-like behavior showed a lower score of MMP-9 KO female mice compared to WT female (*p* < 0.01) and MMP-9 KO male mice (*p* < 0.05, Fig. 5B). Two-way ANOVA revealed a significant effect of sex [F(1,68) = 6.8, *p* < 0.05] and the genotype [F(1,68) = 11.0, *p* < 0.01]. Male and female MMP-9 OE mice and their respective wildtype mice did not significantly differ (Fig. 5D).

### Neuroplasticity markers in the hippocampus of MMP-9 KO and OE mice

**Fig. 6:**
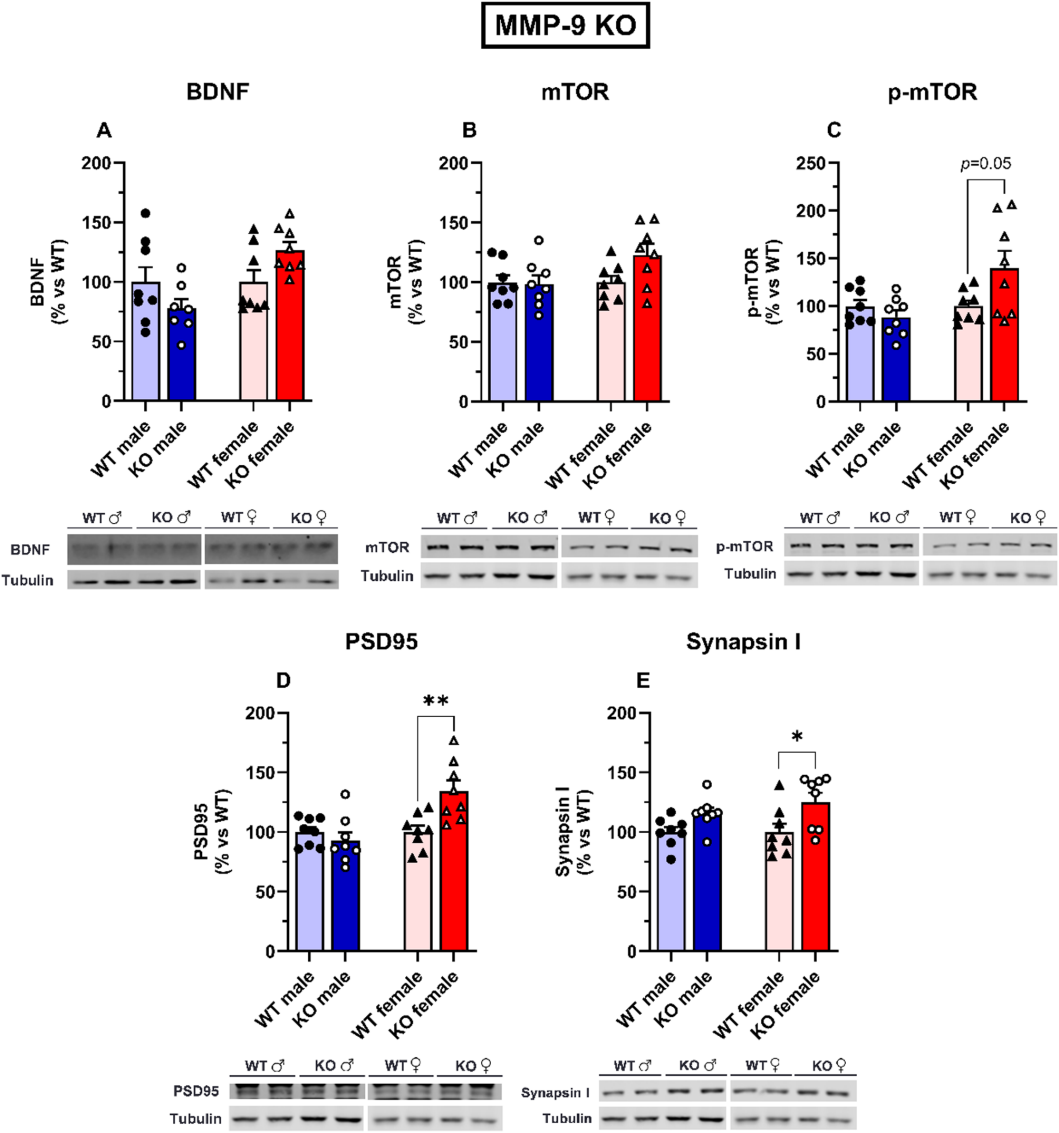
Neuroplasticity markers expression in the hippocampus of male and female MMP-9 KO mice. BDNF (**A**), mTOR (**B**), phospho-mTOR (**C**), PSD95 (**D**), and synapsin I (**E**) expression in MMP-9 KO mice and their corresponding wild-type counterparts. Representative western blot bands are shown. The results are expressed as percentages *versus* the WT group and as the means ± S.E.M.s. Two-way ANOVA followed by a Tukey’s *post hoc* test. *p < 0.05, **p < 0.01. *n* = 7–8 animals per group. BDNF: brain-derived neurotrophic factor; mTOR: mammalian target of rapamycin; PSD95: postsynaptic density 95 protein; WT: wild-type; KO: MMP-9 knockout

In the hippocampus, MMP-9 KO male and female mice did not show differences in BDNF expression levels compared with their WT counterparts (Fig. 6A). Compared with their WT counterparts, the female MMP-9 KO group presented a trend towards increased p-mTOR (*p* = 0.05, Fig. 6C), and increased PSD95 (*p* < 0.01, Fig. 6D) and synapsin l (*p* < 0.05, Fig. 6E) expression levels. No significant differences were observed in the mTOR levels in the male and female MMP-9 KO mice (Fig. 6B). Two-way ANOVA revealed a significant effect of sex [F(1,27) = 6.5, *p* < 0.05] and the interaction sex × genotype [F(1,27) = 6.5, *p* < 0.05] on BDNF values. Two‐way ANOVA revealed a significant effect of sex [F(1,28) = 6.1, *p* < 0.05] and the interaction of genotype × sex [F(1,28) = 6.1, *p* < 0.05] on phospho-mTOR values. Two‐way ANOVA revealed significant effects of sex [F(1,28) = 10.0, *p* < 0.01], genotype [F(1,28) = 4.2, *p* < 0.05] and the interaction of genotype × sex [F(1,28) = 10.0, *p* < 0.01] on PSD95 values. Two‐way ANOVA revealed a significant effect of genotype [F(1,28) = 11.1, *p* < 0.01] on synapsin I values.

**Fig. 7:**
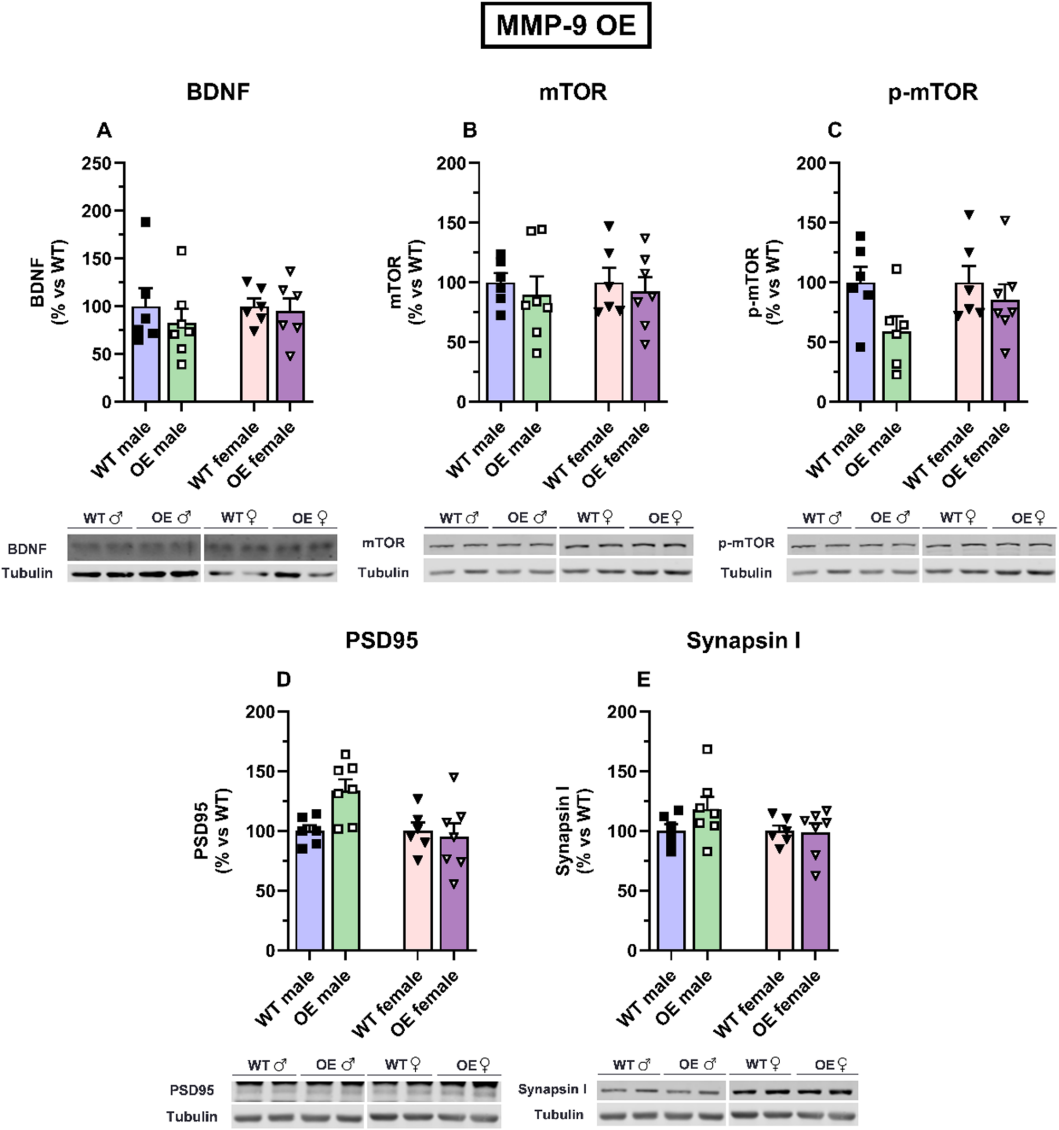
Neuroplasticity markers expression in the hippocampus of male and female MMP-9 OE mice. BDNF (**A**), mTOR (**B**), p-mTOR (**C**), PSD95 (**D**), and synapsin I (**E**) expression in MMP-9 OE mice and their corresponding wild-type counterparts. Representative western blot bands are shown. The results are expressed as percentages *versus* the WT group and as the means ± S.E.M.s. Two-way ANOVA followed by a Tukey’s *post hoc* test. *n* = 6–7 animals per group. BDNF: brain-derived neurotrophic factor; mTOR: mammalian target of rapamycin; PSD95: postsynaptic density 95 protein; WT: wild-type mice; OE: MMP-9-overexpressing mice

The hippocampal levels of BDNF (Fig. 7A), mTOR (Fig. 7B), p-mTOR (Fig. 7C), PSD95 (Fig. 7D), and synapsin I (Fig. 7E) were not significantly different in male and female MMP-9 OE mice compared with their WT counterparts. Two-way ANOVA revealed a significant effect of genotype [F(1,21) = 4.4, *p* < 0.05] on phosphorylated mTOR expression levels. Two‐way ANOVA revealed a significant effect of sex [F(1,22) = 4.9, *p* < 0.05] and the interaction of genotype × sex [F(1,22) = 4.9, *p* < 0.05] on PSD95 expression levels.

## Discussion

The present study focused on the behavioral and molecular parameters of male and female transgenic MMP-9 KO and OE mice. The overexpression of a human transgene in the MMP-9 OE mouse line results in higher gelatinolytic activity in different brain areas such as the hippocampus and cortex, as observed in in vitro studies. In addition, this overexpressed human MMP-9 increases the release of the soluble amyloid precursor protein α (sAPPα) in those brain areas [37].

In our study, we did not observe any statistically significant differences in innate anxiety in male MMP-9 KO mice, which is consistent with previous studies that reported no alterations in anxiety in MMP-9 KO mice [42–44]. Interestingly, female MMP-9 KO mice presented increased innate anxiety, but only in the open field test. This finding might be partially attributed to the lower baseline anxiety levels observed in female wild-type mice than in males, as reported in previous studies in naïve rats [45–47]. The results obtained in the open field test suggest a sex-dependent effect on certain manifestations of innate anxiety in MMP-9 KO mice, which has not been previously described, as other studies present pooled data from both male and female mice [44]. The differences observed in the open field, the elevated plus maze and the light-dark box tests can be attributed to the variability in emotional reactivity which can be associated with genetic and environmental influences. Previous reports indicate the existence of an inter-individual phenotypic variation in different behavioral tests, which can be increased by subjecting the animals to a battery of different behavioral tests [48].

In contrast to the more anxious phenotype observed in the open field test in female MMP-9 KO mice, female MMP-9 OE mice displayed lower innate anxiety which was more evident in the elevated plus maze test. This finding contrasts with previous results in which no differences in innate anxiety were observed in pooled male and female MMP-9 OE mice [37]. Some studies have suggested a link between MMP-9 levels and the basal anxious phenotype in female mice of various strains. For example, female C57BL/6J mice, which exhibit higher basal MMP-9 brain levels, show lower anxiety in the elevated plus-maze test than other strains with lower MMP-9 brain levels and greater anxiety [49]. The lower level of innate anxiety observed in our female MMP-9 OE mice aligns with these findings [49]. Furthermore, increased resilience to anxiety has been reported in female mice in various depression models, such as chronic corticosterone exposure [50, 51] and acute lipopolysaccharide administration [52]. Taken together, these results suggest that MMP-9 plays a sex-dependent role in anxiety.

In male mice, the overexpression of MMP-9 led to increased conflict-based anxiety, which is consistent with the increased anxiety levels observed in the novelty-suppressed feeding test in animal models of depression, such as the chronic corticosterone [25] and obesity [53] models, both of which are characterized by elevated MMP-9 brain levels. However, female MMP-9 OE mice appeared to be protected from developing this conflict-based phenotype. These results support the involvement of MMP-9 in anxiety-related disorders, such as post-traumatic stress disorder [54].

With respect to depressive-like behaviors, female MMP-9 KO mice exhibited decreased behavioral despair, which is consistent with the antidepressant effects observed in various animal models following MMP-9 inhibition [55–57]. Interestingly, this reduced behavioral despair was not observed in male MMP-9 KO mice. Additionally, our MMP-9 OE mice did not show alterations in behavioral despair, which contrasts with the depressive-related behavior typically associated with increased MMP-9 expression and activity in this paradigm [20, 25]. The lack of differences in our transgenic mice may be due to compensatory changes, which are common in constitutive transgenic mice [7].

Depressive disorders are often associated with impaired social functioning [58]. In our study, the overexpression or the deletion of MMP-9 did not affect social interaction parameters. However, MMP-9 KO mice presented a statistically significant genotype effect indicating increased sociability, which may suggest a resilient or less vulnerable phenotype regarding depressive-like manifestations. Other studies report no differences in social behavior in MMP-9 KO mice [43]. These discrepancies may arise from differences in the social interaction protocols used, or the existence of compensatory changes in the studies using constitutive transgenic mice. Several studies in animal models which present high MMP-9 levels suggest that the normalization or reduction of MMP-9 levels can improve sociability [59, 60]. Moreover, transient overexpression of nectin-3, a proteolytic target of MMP-9, is able to reverse stress-induced social deficits [26].

Furthermore, it is important to note that depressive symptomatology is often accompanied by cognitive deficits, both in humans [61, 62] and in animal models of depression [21]. In this study, the deletion of overexpression of MMP-9 did not have an impact on working memory. These results contrast with the negative correlation between hippocampal MMP-9 levels and working memory in male rats [63].

The variability observed in the basal levels of anxiety and depressive-like behaviors, particularly in the elevated plus maze and novelty suppressed feeding tests, may be linked to the genetic background of the transgenic mouse lines used in this study. Specifically, the MMP-9 KO line has a B6;129 genetic origin, while the MMP-9 OE line originates from a B6;DBA background. Although both lines have been crossed with a C57BL/6 background for more than ten generations to minimize genetic differences, the genetic background could still impact behavioral parameters, such as innate anxiety. For example, the 129 strain exhibits a high anxiety-like trait, while the DBA strain displays a moderate anxiety-like trait [64]. However, these differential anxiety responses are not observed in other behavioral tests, such as the open field and light-dark box tests. Furthermore, other experimental factors, including differences between experimenters, the time of day [40], and seasonal changes [65], could also contribute to the behavioral variability.

Our z-score analysis of the anxiety and the depressive-like phenotype shows a marked sex-dependent effect in depressive-like behavior associated to the deletion of MMP-9, while the overexpression of MMP-9 showed a sex-dependent effect on anxiety. Together these findings show a phenotype resilient to depressive-like behavior in MMP-9 OE female mice, which agree with the antidepressant-like effect displayed following MMP-9 inhibition [55–57]. In addition, a phenotype resilient to anxiety is observed in female MMP-9 OE mice, that aligns with previous findings [49].

Depressive disorders are associated with changes in neuroplasticity markers in brain regions such as the hippocampus and prefrontal cortex, which are correlated with symptom severity. Treatment with antidepressant drugs can reverse these changes in neuroplasticity [28]. In this study, we examined neuroplasticity markers associated with depression and the mechanism of action of antidepressant drugs in the hippocampus. Our findings revealed that female MMP-9 KO mice presented increased levels of PSD95 and synapsin l, and a trend toward increased mTOR pathway activation, whereas male MMP-9 KO mice did not show differences. These findings suggest that MMP-9 deletion affects specifically to female and not male mice on depression-related neuroplasticity markers. Several murine models of depression show reduced hippocampal levels of mTOR, PSD95, and synapsin l, which are restored by antidepressant drug treatments, such as serotonin selective reuptake inhibitors (SSRIs) [66–68], ketamine [69, 70], and others [71, 72]. In fact, antidepressant drugs such as fluoxetine and paroxetine normalize mTOR and PSD95 levels in the hippocampus in chronic unpredictable mild stress [66] and chronic social defeat stress models [67]. Thus, the increased expression of these neuroplasticity markers in our MMP-9 KO female mice support the lower depressive-like behavior obtained in the z- score, pointing to a resilient phenotype for depressive-like manifestations in female mice with MMP-9 deletion.

In MMP-9 OE mice, we did not observe changes in hippocampal neuroplasticity markers. This would correlate with the lack of differences observed in the depressive-like behavioral parameters in these MMP-9 OE transgenic mice, as observed in the z-score analysis. In this sense, although the reduction of BDNF [73], mTOR pathway activation [74] has been associated to an increased anxiety-like behavior, other hippocampal neural markers might be correlated with the lower anxiety elicited by female MMP-9 OE mice. Moreover, changes in neuroplasticity markers in other brain areas important in anxiety such as the amygdala [29], may be having a more relevant role in this behavioral effect.

The existence of compensatory mechanisms in our transgenic animals, affecting to the activity of other metalloproteinases, cannot be discarded. In this regard, an increase in the expression and activity of MMP-9 has been described in an MMP-2 knockout mouse [75], while no changes in MMP-2 expression have been reported in animals overexpressing MMP-9 [76].

As above described, MMP-9 is consistently associated with anxious and depressive disorders in animal models [25] and in human peripheral samples [12, 14, 16, 77], while the role of other metalloproteinases, such as MMP-2, is not clear. Our previous results show an increase in MMP-9 expression in hippocampal and cortical areas in an animal model of depression, while MMP-2 levels did not differ [25].

Our study highlights the impact of MMP-9 expression levels on anxiety and depressive-like behaviors. A limitation of our research is the lack of consideration of the estrous cycle in female animals. Although monitoring the estrous cycle could provide valuable information about MMP-9 expression in these animals, we did not perform vaginal smears to prevent exposing female mice to extra stress conditions. The z-score analysis of our behavioral results minimizes the potential effect of the estrous stage in the anxious and depressive-like behavior. Another limitation is the effect of acute stress exposure in the protein expression of the neuroplasticity markers studied, compared to naïve animals. This effect might not be very relevant at the time point studied, as we have evaluated protein and not mRNA expression levels. In this sense, none of the proteins analyzed in our study appear to be affected by acute stress [78].

## Conclusions

Our study highlights the sex-dependent role of MMP-9 in anxiety and depression. However, the regulation of MMP-9 levels does not appear to be the sole factor driving the modulation of anxiety and depression-like manifestations. These findings emphasize the importance of including female subjects in preclinical and clinical studies of depression and anxiety, as the incidence of these disorders is generally higher in women than in men.

## Electronic supplementary material

Below is the link to the electronic supplementary material.

Supplementary Material 1

## Data Availability

The data that support the findings of this study are available upon request from the corresponding author.

## Abbreviations

BDNF: Brain-derived neurotrophic factor
EPM: Elevated plus maze
KO: Knockout transgenic mouse
LDB: Light‒dark box test
MDD: Major depressive disorder
MMP: Matrix metalloproteinase
mTOR: Mammalian target of rapamycin
NSF: Novelty-suppressed feeding test
OE: Overexpression transgenic mouse
OFT: Open-field test
PSD95: Postsynaptic density protein 95
SIT: Social interaction test
TST: Tail suspension test
WT: Wild-type mouse
