# Supplementary material for: Sex differences in the modulation of anxiety- and depression-like behaviors by matrix metalloproteinase-9 expression levels in mice
Source: Biol Sex Differ. 2025 May 22;16:34. doi: 10.1186/s13293-025-00716-5 (PMC12096558; doi:10.1186/s13293-025-00716-5)
Supplement: Supplementary file 1 — Supplementary Material 1 [file 13293_2025_716_MOESM1_ESM.pdf]

Sex Differences in the Modulation of Anxiety- and Depression-like Behaviors by Matrix Metalloproteinase-9 Expression Levels in Mice

Júlia Senserrich[1,2,*], Elena Castro[1,2,3,*], Eva Florensa-Zanuy[1,2], Álvaro Díaz[1,2,3], Ángel Pazos[1,2,3], Albert Adell[1,2], Athina Tzinia[4] and Fuencisla Pilar-Cuéllar[1,2,3,&]

SUPPLEMENTARY MATERIAL

Table S1. Primary antibodies used for protein detection by Western Blot, with their corresponding dilution, reference, supplier, Research Resource Identifiers (RRIDs), and references. BDNF: brain-derived neurotrophic factor; mTOR: mammalian target of rapamycin; PSD95: postsynaptic density protein 95; rb: rabbit; m: mouse; g: goat; RRID: research resource identifier.

ANTIBODY	DILUTION	REFERENCE	SUPPLIER	RRIDs	REFERENCES
β-tubulin III (rb, m)	1:20000	T2200/T8660	Sigma–Aldrich	AB_262133/ AB_477590	[1], [2], [3], [4], [5], [6]
BDNF (rb)	1:500	ab108319	Abcam	AB_10862052	[7], [8], [9], [10], [11]
mTOR (m)	1:1000	#4517	Cell Signaling Technology	AB_1904056	[12], [13], [14], [15]
Phospho-mTOR (Ser2448) (rb)	1:1000	#2971	Cell Signaling Technology	AB_330970	[12], [13], [16], [17], [18], [19]
PSD95 (g)	1:200	sc-8575	Santa Cruz Biotechnology	AB_2092357	[18], [20], [21]
Synapsin Ia/b (m)	1:200	sc-390867	Santa Cruz Biotechnology	AB_3678685	[22], [23]

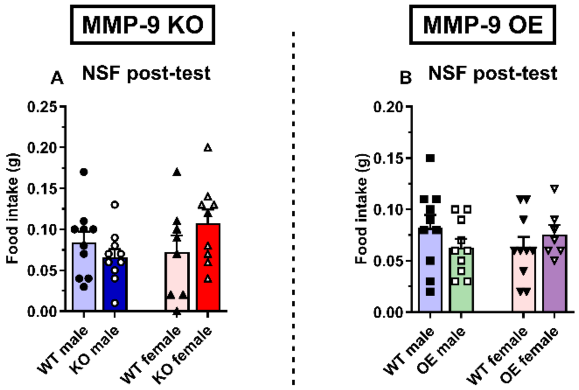

Figure S1. Post-test of the novelty-suppressed feeding test. Food consumed by MMP-9 KO (A) and MMP-9 OE (B) male and female mice in their homecage following the novelty-suppressed feeding test. The data are expressed as the means ± SEMs. Two-way ANOVA followed by an uncorrected Fisher's LSD post hoc test. n = 7–10 animals per group. WT: wild-type mice; KO: MMP-9 knockout mice; OE: MMP-9-overexpressing mice.

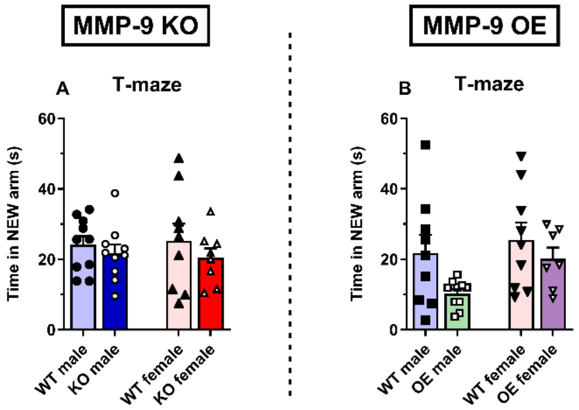

Figure S2. Spatial working memory in MMP-9 KO (A) and MMP-9 OE (B) male and female mice assessed by the time spent in the novel arm in the T-maze. The results are expressed as the means ± S.E.M.s. Two-way ANOVA followed by a Tukey's *post hoc* test. n= 8 - 10 animals per group. WT: wild-type mice; KO: MMP-9 knockout mice; OE: MMP-9-overexpressing mice.

BIBLIOGRAPHY

1. Friedman TN, Lamothe SM, Maguire AD, Hammond T, Tenorio G, Hilton BJ, et al. Plasticity of Mouse Dorsal Root Ganglion Neurons by Innate Immune Activation Is Influenced by Electrophysiological Activity. J Neurochem. 2025; doi: 10.1111/jnc.16292.
2. Sztachera M, Wendlandt-Stanek W, Serwa RA, Stanaszek L, Smuszkiewicz M, Wronka D, Piwecka M. Interrogation of RNA-bound proteome with XRNAX illuminates molecular alterations in the mouse brain affected with dysmyelination. Cell Rep. 2025; doi: 10.1016/j.celrep.2024.115095.
3. Cachán-Vega C, Vega-Naredo I, Potes Y, Bermejo-Millo JC, Rubio-González A, García-González C, et al. Chronic Treatment with Melatonin Improves Hippocampal Neurogenesis in the Aged Brain and Under Neurodegeneration. Molecules. 2022; doi: 10.3390/molecules27175543.
4. Amadeo A, Pizzi S, Comincini A, Modena D, Calogero AM, Madaschi L, et al. The Association between α-Synuclein and α-Tubulin in Brain Synapses. Int J Mol Sci. 2021; doi: 10.3390/ijms22179153.
5. Miyoshi K, Kasahara K, Miyazaki I, Asanuma M. Lithium treatment elongates primary cilia in the mouse brain and in cultured cells. Biochem Biophys Res Commun. 2009; doi: 10.1016/j.bbrc.2009.08.099.
6. Asaka Y, Jugloff DG, Zhang L, Eubanks JH, Fitzsimonds RM. Hippocampal synaptic plasticity is impaired in the Mecp2-null mouse model of Rett syndrome. Neurobiol Dis. 2006; doi: 10.1016/j.nbd.2005.07.005.

7. Chavez-Valdez R, Martin LJ, Razdan S, Gauda EB, Northington FJ. Sexual dimorphism in BDNF signaling after neonatal hypoxia-ischemia and treatment with necrostatin-1. Neuroscience. 2014 Feb 28;260:106-19. doi: 10.1016/j.neuroscience.2013.12.023.
8. Rogers J, Vo U, Buret LS, Pang TY, Meiklejohn H, Zeleznikow-Johnston A, et al. Dissociating the therapeutic effects of environmental enrichment and exercise in a mouse model of anxiety with cognitive impairment. Transl Psychiatry. 2016; doi: 10.1038/tp.2016.52.
9. Xu H, Zhang Y, Zhang F, Yuan SN, Shao F, Wang W. Effects of Duloxetine Treatment on Cognitive Flexibility and BDNF Expression in the mPFC of Adult Male Mice Exposed to Social Stress during Adolescence. Front Mol Neurosci. 2016; doi: 10.3389/fnmol.2016.00095.
10. Yao W, Lin S, Su J, Cao Q, Chen Y, Chen J, et al. Activation of BDNF by transcription factor Nrf2 contributes to antidepressant-like actions in rodents. Transl Psychiatry. 2021; doi: 10.1038/s41398-021-01261-6.
11. Zhang K, Wang F, Zhai M, He M, Hu Y, Feng L, et al. Hyperactive neuronal autophagy depletes BDNF and impairs adult hippocampal neurogenesis in a corticosterone-induced mouse model of depression. Theranostics. 2023; doi: 10.7150/thno.81067.
12. Gordillo-Salas M, Pilar-Cuéllar F, Auberson YP, Adell A. Signaling pathways responsible for the rapid antidepressant-like effects of a GluN2A-preferring NMDA receptor antagonist. Transl Psychiatry. 2018; doi: 10.1038/s41398-018-0131-9.
13. Pilar-Cuellar F, Castro E, Bretin S, Mocaer E, Pazos Á, Díaz Á. S 47445 counteracts the behavioral manifestations and hippocampal

neuroplasticity changes in bulbectomized mice. Prog Neuropsychopharmacol Biol Psychiatry. 2019; doi: 10.1016/j.pnpbp.2019.04.005.

14. Sanchez-Bezanilla S, Beard DJ, Hood RJ, Åberg ND, Crock P, Walker FR, et al. Growth Hormone Increases BDNF and mTOR Expression in Specific Brain Regions after Photothrombotic Stroke in Mice. Neural Plast. 2022; doi: 10.1155/2022/9983042.

15. Kommaddi RP, Gowaikar R, P A H, Diwakar L, Singh K, Mondal A. Akt activation ameliorates deficits in hippocampal-dependent memory and activity-dependent synaptic protein synthesis in an Alzheimer's disease mouse model. J Biol Chem. 2024; doi: 10.1016/j.jbc.2023.105619.

16. Wallin DJ, Zamora TG, Alexander M, Ennis KM, Tran PV, Georgieff MK. Neonatal mouse hippocampus: phlebotomy-induced anemia diminishes and treatment with erythropoietin partially rescues mammalian target of rapamycin signaling. Pediatr Res. 2017; doi: 10.1038/pr.2017.88.

17. Wahl D, Solon-Biet SM, Wang QP, Wali JA, Pulpitel T, Clark X, et al. Comparing the Effects of Low-Protein and High-Carbohydrate Diets and Caloric Restriction on Brain Aging in Mice. Cell Rep. 2018, doi: 10.1016/j.celrep.2018.10.070.

18. Cabanu S, Pilar-Cuéllar F, Zubakina P, Florensa-Zanuy E, Senserrich J, Newman-Tancredi A, Adell A. Molecular Signaling Mechanisms for the Antidepressant Effects of NLX-101, a Selective Cortical 5-HT1A Receptor Biased Agonist. Pharmaceuticals (Basel). 2022; doi: 10.3390/ph15030337.

19. Wang YL, Wang JG, Guo S, Guo FL, Liu EJ, Yang X, et al. Oligomeric β-Amyloid Suppresses Hippocampal γ-Oscillations through Activation of the mTOR/S6K1 Pathway. Aging Dis. 2023; doi: 10.14336/AD.2023.0123.
20. Amar F, Sherman MA, Rush T, Larson M, Boyle G, Chang L, et al. The amyloid-β oligomer Aβ*56 induces specific alterations in neuronal signaling that lead to tau phosphorylation and aggregation. Sci Signal. 2017; doi: 10.1126/scisignal.aal2021.
21. Tao W, Yao G, Yue Q, Xu C, Hu Y, Cheng X, et al. 14-3-3ζ Plays a key role in the modulation of neuroplasticity underlying the antidepressant-like effects of Zhi-Zi-Chi-Tang. Phytomedicine. 2023; doi: 10.1016/j.phymed.2023.154888.
22. Gąssowska-Dobrowolska M, Cieślik M, Czapski GA, Jęśko H, Frontczak-Baniewicz M, Gewartowska M, et al. Prenatal Exposure to Valproic Acid Affects Microglia and Synaptic Ultrastructure in a Brain-Region-Specific Manner in Young-Adult Male Rats: Relevance to Autism Spectrum Disorders. Int J Mol Sci. 2020; doi: 10.3390/ijms21103576.
23. Cieślik M, Gassowska-Dobrowolska M, Zawadzka A, Frontczak-Baniewicz M, Gewartowska M, Dominiak A, et al. The Synaptic Dysregulation in Adolescent Rats Exposed to Maternal Immune Activation. Front Mol Neurosci. 2021; doi: 10.3389/fnmol.2020.555290.
